# Value of subcarinal lymph node dissection in esophageal cancer surgery: A case-control study

**DOI:** 10.1097/MD.0000000000031593

**Published:** 2022-10-28

**Authors:** Bindong Xu, Hao Chen, Qiang Zhang, Pengfei Chen, Qiuxia Liu, Mingyu Chen

**Affiliations:** a Department of Thoracic and Cardiovascular Surgery of the Affiliated Hospital of Putian University, Putian, Fujian, China.

**Keywords:** dissection, esophageal cancer, subcarinal lymph node

## Abstract

We investigated the value of subcarinal lymph node dissection in esophageal cancer surgery. Altogether, 240 patients with esophageal cancer admitted to our department between June 2012 and January 2016 were prospectively assigned to an experimental group (subcarinal lymph node dissection group, n = 120 cases) and a control group (uncleaned group, n = 120 cases). The number of subcarinal lymph nodes and the rate of subcarinal lymph node metastasis were determined, and the factors influencing subcarinal lymph node metastasis were analyzed using logistic regression in the experimental group. The operation time, postoperative complications, intraoperative blood loss, postoperative hospital stay, total postoperative hospital cost, and 5-year survival rate were compared between the 2 groups. In the experimental group, an average of 6.03 subcarinal lymph nodes were dissected, and the lymph node metastasis rate was 18.33%. The subcarinal lymph node metastasis rate in the experimental group was related to the size of the subcarinal lymph nodes, depth of tumor invasion, and tumor location. The 5-year survival rate was higher in the experimental group than in the control group (44.2% vs 30.0%, *χ*^2^ = 6.407, *P* = .04). The subcarinal lymph node metastasis rate in patients with esophageal cancer is high. Patients with mid-thoracic esophageal cancers that infiltrate beyond the esophageal muscle layer with subcarinal lymph node size > 1.0 cm should undergo lymph node resection, despite increased operation time, incidence of postoperative pulmonary infection, hospitalization time, and total postoperative cost; lymph node resection may improve the 5-year survival rate.

## 1. Introduction

Esophageal cancer and esophagogastric junction cancer are among the most common digestive tract malignancies, accounting for 310,400 new cases worldwide yearly,^[1]^ of which > 50% are noted in China.^[2]^ Similar to other gastrointestinal malignancies, lymph node metastasis is an important pathway for distant metastasis of esophageal cancer.^[3]^ Surgical treatment of esophageal cancer requires not only complete resection of the diseased esophagus but also systematic resection. Mediastinal lymph nodes are routinely resected, and the subcarinal lymph nodes represent the extent of mediastinal lymph node resection. Therefore, the subcarinal lymph nodes are within the scope of routine dissection. However, subcarinal lymphadenopathy may also be caused by benign chest diseases such as lung infection and silicosis. Moreover, excessive subcarinal lymph node dissection increases surgical trauma and pulmonary infection and reduces the short-term quality of life of patients postoperatively.^[4]^ Dissection of subcarinal lymph nodes has always been problematic for esophageal surgeons. Therefore, this study aimed to investigate subcarinal lymph node dissection to understand its value in esophageal cancer surgery.

## 2. Materials and Methods

### 2.1. Patient characteristics

In total, 240 patients (161 men, 79 women; age 46–81 [64.69 ± 7.259] years) with esophageal and esophagogastric junction cancer who received surgery in our department from June 2012 to January 2015 were selected. Of all cases, 25, 145, and 54 were localized in the upper, middle, and lower thoracic esophageal junction, respectively, and 16 in the esophagogastric junction. In the postoperative pathological diagnosis, all esophageal carcinomas were squamous cell carcinomas, and all esophagogastric junction carcinomas were adenocarcinomas. In this study population, 53, 10, 48, 9, 120, 59, 44, and 17 tumors were in stages Tis/T1, T2, T3, T4, N0, N1, N2, and N3, respectively, based on the 2009 International Union Against Cancer and American Joint Committee on Cancer standards for pathological grading and staging.^[5]^ Furthermore, 82, 99, and 49 were well-differentiated, moderately differentiated, and poorly differentiated cancers, respectively. According to stage, 64, 76, 82, and 18 cases were in stages I, II, III, and IV, respectively. Tumor length was ≤3 cm, 3 to 5 cm, and >5 cm in 53, 45, and 22 cases, respectively. The study protocol was approved by the Ethics Committee of our hospital (approval number, 202039). All patients enrolled in the study provided written informed consent.

### 2.2. Experimental design

We prospectively assigned patients into an experimental group (subcarinal lymph node dissection group, n = 120 cases) and a control group (uncleaned group, n = 120 cases).

The inclusion criteria were pathological diagnosis of malignant tumor by electronic gastroscope biopsy with clinicopathological stage cT1-3N0-3M0; computed tomography (CT) revealing no metastasis; no radiotherapy or chemotherapy before operation; and complete postoperative follow-up treatment.

The exclusion criteria were small cell carcinoma upon preoperative electronic gastroscopic biopsy; preoperative severe cardiopulmonary disease or inability to tolerate surgery; concurrent presence of other malignant tumors or previous history of malignant tumors; preoperative radiotherapy and/or chemotherapy; and incomplete postoperative follow-up data.

### 2.3. Surgical technique

Thoracic and upper thoracic esophageal cancers were treated using thoracic laparoscopy combined with lower 3-field radical esophagectomy, and lower thoracic esophageal and esophagogastric junction cancers were treated using right thoracoabdominal esophagectomy.

In the experimental group, lymph nodes around the bilateral recurrent laryngeal nerve, middle esophagus, lower esophagus, and below the carina were dissected. In the control group, lymph nodes around the bilateral recurrent laryngeal nerve, middle esophagus, and lower esophagus were dissected. The postoperative management was the same for all patients. All patients who required postoperative radiotherapy and/or chemotherapy received the same regimen.

### 2.4. Data collection

The number of lymph nodes dissected and the rate of lymph node metastasis in the 2 groups of patients were documented. The number of subcarinal lymph nodes dissected and the rate of lymph node metastasis in the experimental group were determined. The operation time, intraoperative blood loss, incidence of postoperative complications, postoperative hospitalization time, total postoperative hospitalization costs, mortality, and postoperative 5-year survival rates of the 2 groups were compared.

### 2.5. Follow-up methods

Follow-up was once every 3 months for 1 year postoperatively; once every 6 months for 1 to 2 years postoperatively; and once a year for 3 to 5 years postoperatively. Detailed medical history; physical examination; investigations for the full set of tumor markers; direct enhanced CT of the brain, neck, chest, and upper abdomen; and whole-body bone imaging were checked during follow-up. Follow-up endpoint was death.

### 2.6. Statistical analysis

The SPSS version 25.0 software (IBM, Armonk, NY) was used for statistical analysis; measured data are expressed as mean ± standard deviation, and the comparison between the 2 groups was performed using the t-test. Enumerated data were compared between the groups using the chi-square test and are presented as frequencies (percentages). Logistic regression analysis was used for the multivariate analysis. Statistical significance was set at *P* < .05.

## 3. Results

No significant differences were noted in sex, age, tumor location, tumor length, postoperative pathological staging, or postoperative tumor differentiation between the 2 groups. All patients in the 2 groups successfully completed the operation, and no deaths occurred during or immediately after the operation (Table 1).

**Table 1:** Comparison of general data of the 2 groups of patients.

Project	Experimental group	Control group	t/χ^2^ value	*P* value
Sex			2.283	.13
MaleWW	75	86		
Female	45	34		
Age (yrs)	65.16 ± 7.314	64.22 ± 7.205	0.996	.32
Tumor site (case)			2.412	.49
Upper chest	15	10		
Middle chest	69	76		
Lower chest	26	28		
Esophagogastric junction cancer	10	6		
Postoperative pathological staging (cases)			2.190	.53
I	27	37		
II	40	36		
III	43	39		
IVA	10	8		
Degree of tumor differentiation (cases)			1.896	.39
Well differentiated	36	46		
Moderately differentiated	52	47		
Poorly differentiated	32	27		

### 3.1. Intraoperative lymph node dissection

A total of 7916 lymph nodes were dissected in both groups (mean, 32.98; lymph node metastasis rate, 5.99%). In the experimental group, 724 subcarinal lymph nodes were dissected (mean, 6.03; lymph node metastasis rate 1.66%).

### 3.2. Comparison of the perioperative conditions

The operation time of the experimental group was longer than that of the control group at 271.76 ± 69.488 versus 253.05 ± 68.694 minutes (t = 2.097, *P* = .04). The intraoperative blood loss was 230.71 ± 107.893 mL, which was higher than that of the control group, 202.13 ± 84.875 mL (t = 2.281, *P* = .02). The incidence of postoperative pulmonary infective complications was 40.00%, which was 25.83% higher than that of the control group (*χ*^2 ^= 5.453, *P* = .02). The postoperative hospital stay and total postoperative cost were higher in the experimental group than in the control group (19.72 ± 9.413 vs 17.64 ± 7.745 days, t = 2.061, *P* = .04 and 72718.4 ± 13725.27 vs 69437.7 ± 10152.64 yuan, t = 2.105, *P* = .04, respectively) (Table 2).

**Table 2:** Comparison of the perioperative conditions in the 2 groups of patients.

Project	Experimental group	Control group	t/χ^2^ value	*P* value
Operation time (min)	271.76 ± 69.488	253.05 ± 68.694	2.097	.04
Intraoperative blood loss (mL)	230.71 ± 107.893	202.13 ± 84.875	2.281	.02
Postoperative complications (cases)				
Lung infection	48	31	5.453	.02
Anastomotic fistula	21	10	4.482	.03
Surgical site infection	10	8	0.240	.62
Urinary tract infection	4	5	0.115	.73
Blood system infection	3	2	0.204	.65
Postoperative anastomotic recurrence (cases)	6	12	2.162	.14
Postoperative liver metastasis (cases)	4	6	0.417	.52
Postoperative lung metastasis (cases)	4	3	0.147	.70
Postoperative bone metastasis (cases)	3	1	1.017	.31
Postoperative hospital stay (d)	19.72 ± 9.413	17.64 ± 7.745	2.061	.04
Total cost after surgery (yuan)	72718.4 ± 13725.27	69437.7 ± 10152.64	2.105	.04

### 3.3. Analysis of factors affecting subcarinal lymph node metastasis

Logistic regression analysis revealed that the subcarinal lymph node metastasis rate in the experimental group was related to the subcarinal lymph node size, tumor infiltration depth, tumor location, and tumor length (Table 3).

**Table 3:** Analysis of factors affecting subcarinal lymph node metastasis.

	B	S.E	Wals	df	Sig.	Exp (B) (95%CI)
Tumor site	−1.021	0.505	4.085	1	0.043	0.360 (0.134–0.970)
Subcarinal lymph node size	1.440	0.730	3.892	1	0.049	4.222 (1.009–17.659)
Depth of tumor invasion	0.686	0.337	4.150	1	0.042	1.986 (1.026–3.844)
Tumor length	1.740	0.523	11.058	1	0.001	5.696 (2.043–15.880)
Constant	−6.800	1.605	17.955	1	0.000	0.001

### 3.4. Comparison of the 5-year survival rate

**Figure 1.**
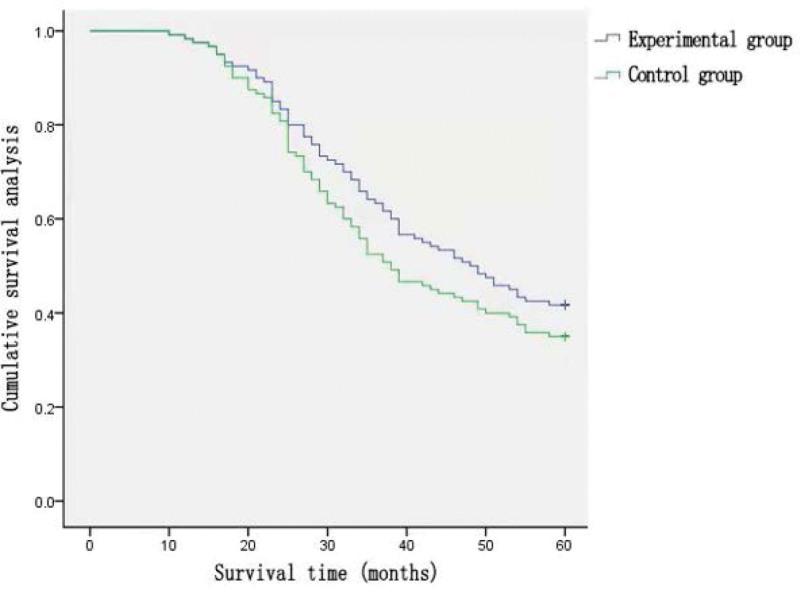
The 5-year survival rate in the experimental group (44.20%) is higher than that in the control group (27.50%).

A total of 240 patients in the 2 groups were followed up; 18 (7.5%) were lost to follow-up. The 5-year survival rate in the experimental group was 44.2%, which was significantly higher than that in the control group (30.0%) (*χ*^2 ^= 6.407, *P* = .04) (Fig. 1).

## 4. Discussion

For esophageal cancer and esophagogastric junction cancer, the distant metastasis routes include lymphatic and hematogenous metastasis, with lymphatic metastasis as the most common route. Cancer cells first enter the submucosal lymph nodes of the esophagus and pass through the muscle layer into the regional lymph nodes that drain the tumor site; moreover, the affected lymph nodes drain the neck, chest, and abdomen. Furthermore, lymph node metastasis is mostly longitudinal^[3]^ and directly affects the prognosis of patients with esophageal cancer^[6]^; it is also one of the main causes of death after esophageal cancer surgery.^[7]^ Subcarinal lymph nodes are important regional lymph nodes in the chest, and their reasonable dissection can help in accurate pathological staging and improve the survival rate of patients postoperatively.^[8–10]^ However, some researchers^[11]^ have reported only a 10.4% rate of metastasis to subcarinal lymph nodes, which was significantly lower than the rate of metastasis to other regional lymph nodes, and the incidence of lung-related complications after postoperative dissection of subcarinal lymph nodes increases significantly. Therefore, routine postoperative dissection of the subcarinal lymph nodes is not recommended for patients with esophageal cancer. In this study, the subcarinal lymph node metastasis rate in the experimental group was 18.33% (22/120), which may be related to the fact that most of the esophageal cancers in this group were squamous cell carcinomas, and most patients visited a doctor when they had difficulty swallowing, which suggested that the disease was in the middle or late stages.

In this study, the pulmonary-related complications in the experimental group were 40%, which was significantly higher than that of the control group; this may be due to damage to the medial border of the left and right main bronchi, left and right bronchial arteries below the carina, and pulmonary plexus branch of the vagus nerve during the dissection of the lymph nodes in this region. Additionally, when the electrocoagulation hook or ultrasonic scalpel is used to resect the lymph nodes in this area, the left and right bronchi may be thermally stimulated, which may lead to an increase in postoperative respiratory secretions.^[12]^ Paroxysmal bronchial smooth muscle contraction occurs, producing an irritating cough. Concurrently, an imbalance of cholinergic receptors can cause the accumulation of alveolar secretions, leading to pulmonary complications such as pulmonary infection. Patients with preoperative chronic bronchial disease could experience respiratory failure and even acute respiratory distress syndrome, which is life-threatening during the procedure. Additionally, bronchial artery damage and bronchial heat stimulation can cause bronchial mucosal ischemia, which negatively affects bronchial mucociliary function, causing difficulty in sputum removal, further aggravating pulmonary infection, increasing operation time, intraoperative blood loss, length of postoperative hospital stay, and total cost of postoperative hospital stay.

Subcarinal lymphadenopathy is not specific to malignancies. Long-term smoking, silicosis, chronic lung disease, and advanced age can lead to enlarged subcarinal lymph nodes. In this study, preoperative chest CT revealed a metastasis rate of esophageal cancer in patients with subcarinal lymph node diameters <1.0 and ≥1.0 cm of 5.41% and 39.13%, respectively. Therefore, when the subcarinal lymph node diameter exceeds 1.0 cm, routine dissection of the lymph nodes in this area is required. However, Shibamoto et al^[13]^ have revealed that the size of the subcarinal lymph nodes cannot be used as a basis for judging whether the cancer has metastasized to the lymph nodes in the region, and that it is not necessary to routinely dissect the regional lymph nodes. After frozen section to confirm metastasis, the lymph nodes may then be resected. Our findings may be related to the fact that most patients in the experimental group in this study were in the middle and late stages of the pathology.

In this study, the subcarinal lymph node metastasis rates of upper, middle, and lower thoracic esophageal, and esophagogastric junction cancers in the experimental group were 7.14%, 38.00%, 1.45%, and 10.00%, respectively. The subcarinal lymph node metastasis rate was significantly higher in esophageal cancer in the mid-thoracic region than in cancers in other parts of the esophagus, which may be related to the fact that mid-thoracic esophageal cancer is the closest to the subcarinal lymph node and is more likely to metastasize to the regional lymph nodes. Furthermore, some studies have demonstrated tumor invasion depth to be an independent risk factor for subcarinal lymph node metastasis. In this study, the logistic regression analysis indicated that Tis/T1 stage esophageal cancer was less likely to cause subcarinal lymph node metastasis. Once the tumor infiltrates beyond the muscle layer, it can invade lymphatic vessels and reach the regional lymph nodes. The cancer may then metastasize to the subcarinal lymph nodes, and the deeper the infiltration, the greater the chance of subcarinal lymph node metastasis.

The subcarinal lymph node metastasis rates of esophageal cancer of tumor length ≤3 cm, 3 to 5 cm, and >5 cm were 18.87%, 8.89%, and 36.36%, respectively, in the experimental group. Therefore, the tumor length is an important factor in lymph node metastasis. It has been suggested that the length of the esophageal tumor is related to subcarinal lymph node metastasis. This is because the longer the esophageal tumor invading the surrounding tissues of the esophagus, the greater likelihood that invasion of the lymphatic vessels in the subcarinal area will occur, resulting in subcarinal lymph node metastasis; this is consistent with the findings of Feng.^[6]^

In the experimental group, the subcarinal lymph node metastasis rates of well-differentiated, moderately differentiated, and poorly differentiated esophageal cancers were 8.13%, 8.02%, and 6.29%, respectively, and the difference was not statistically significant. This suggests that subcarinal lymph node metastasis was not associated with the degree of tumor differentiation because the degree of tumor differentiation only reflects the degree of malignancy of the tumor, and whether subcarinal lymph node metastasis occurs is mainly related to whether the cancer cells invade the lymphatic vessels in this area. Therefore, tumors that are less differentiated do not have a greater chance of invading lymphatic vessels in the region.

The 5-year follow-up rate was 92.5%; 18 patients had anastomotic recurrence, 10 had liver metastasis, 7 had lung metastasis, and 4 had bone metastasis. The 5-year survival rate of the experimental group was 44.2%, which was significantly higher than that of the control group (30.0%). Subcarinal lymph node dissection may improve the 5-year survival rate of patients with esophageal cancer.

### 4.1. Study limitations

This was a single-center study that had a relatively small number of enrolled cases and a short-term follow-up. Thus, the results need to be further validated. The next step is to extend this research to other hospitals and perform a statistical analysis of the 10-year postsurgical survival rate.

In conclusion, the subcarinal lymph node metastasis rate in patients with esophageal cancer was high, and the metastasis rate was related to the size of the subcarinal lymph node, depth of tumor invasion, tumor location, and tumor pathological stage. For patients with mid-thoracic esophageal cancers infiltrating beyond the adventitia of the esophagus and those with subcarinal lymph node size >1.0 cm, routine lymph node dissection in this area should be performed; although this may prolong the operation time, increase the incidence of postoperative pulmonary infection, prolong the hospitalization time, and increase the total postoperative cost, it may improve the 5-year survival rate.

## Acknowledgments

We thank Huang Guozhong (Department of Cardiothoracic Surgery, The Affiliated Hospital of Putian University, Putian Fujian, China) for his assistance with editing this manuscript.

## Author contributions

Study conception and design: Bindong Xu and Hao Chen. Data collection: Bindong Xu, Hao Chen, Qiang Zhang, and Pengfei Chen. Analysis and interpretation of results: Bindong Xu, Hao Chen, Qiuxia Liu, Mingyu Chen and Qiang Zhang. Manuscript preparation: Bindong Xu and Hao Chen. All authors reviewed the results and approved the final version of the manuscript.

**Conceptualization:** Bindong Xu, Hao Chen, Qiang Zhang, Mingyu Chen.

**Data curation:** Bindong Xu, Qiang Zhang, Qiu Xia.

**Formal analysis:** Bindong Xu, Hao Chen, Pengfei Chen, Qiu Xia.

**Investigation:** Bindong Xu, Mingyu Chen.

**Resources:** Hao Chen, Pengfei Chen.

**Software:** Qiang Zhang, Pengfei Chen.

**Writing – original draft:** Bindong Xu.

**Writing – review & editing:** Bindong Xu.
